# Supplementary material for: Progressive overload without progressing load? The effects of load or repetition progression on muscular adaptations
Source: PeerJ. 2022 Sep 30;10:e14142. doi: 10.7717/peerj.14142 (PMC9528903; doi:10.7717/peerj.14142)
Supplement: Supplemental Information 2 — We re-ran all analyses after excluding each participant, one at a time. This assessed the influence of each participant on the estimated treatment effect. There were some instances where individual participants were indeed influential (e.g., MG muscle thickness), but none of these instances were enough to alter our conclusions. [file peerj-10-14142-s002.pdf]

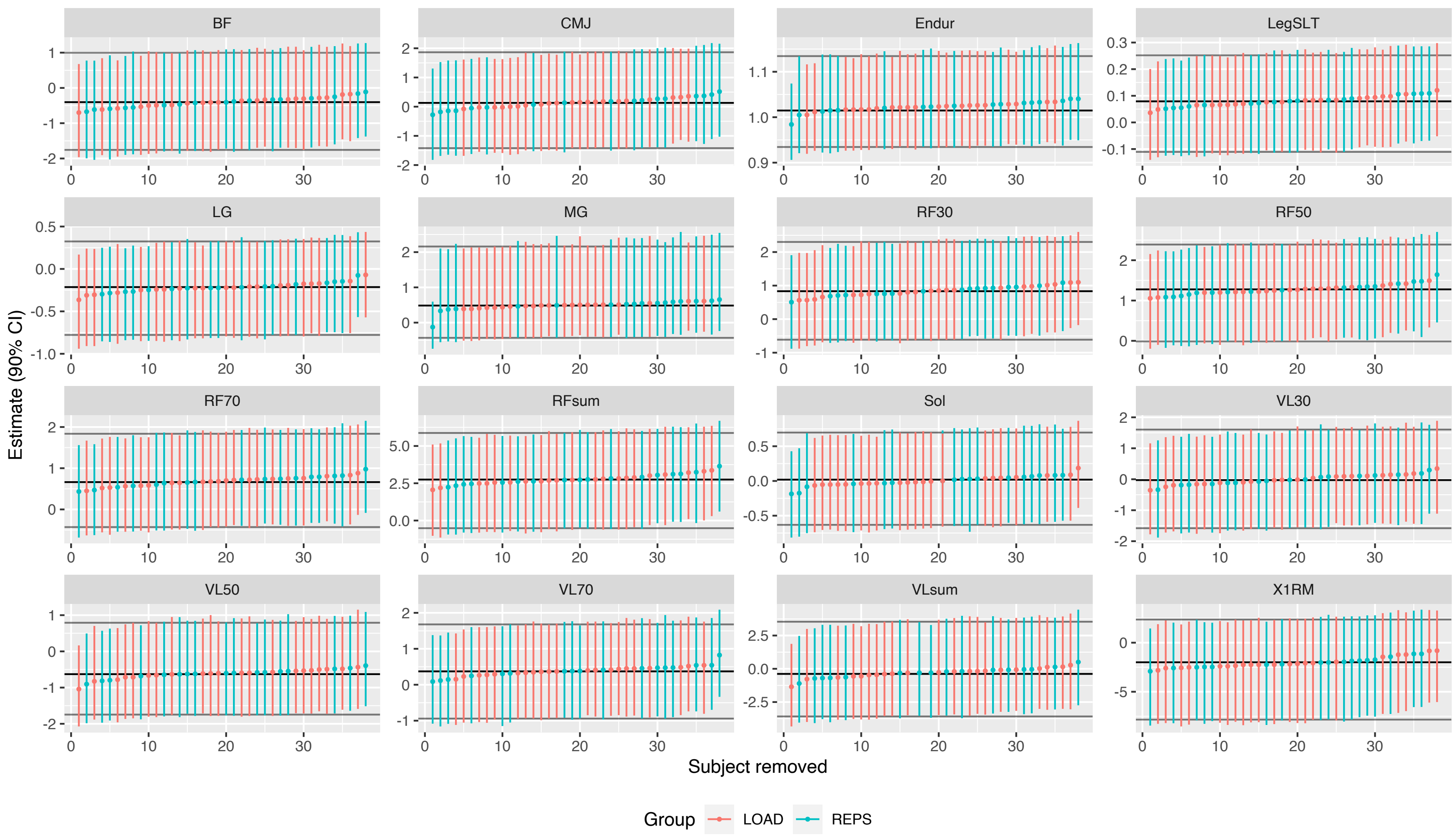

Figure S1. Leave-one-out sensitivity analysis of all outcomes. We re-ran all analyses after excluding each participant, one at a time. This assessed the influence of each participant on the estimated treatment effect. There were some instances where individual participants were indeed influential (e.g., MG muscle thickness), but none of these instances were enough to alter our conclusions.
